# Foods and beverages provided in out of school hours care services: an observational study

**DOI:** 10.1186/s12889-022-12652-9

**Published:** 2022-02-11

**Authors:** Ruth K. Crowe, Yasmine C. Probst, Jennifer A. Norman, Susan E. Furber, Rebecca M. Stanley, Sarah T. Ryan, Cecilia Vuong, Megan L. Hammersley, Karen Wardle, Lisa Franco, Michael W. Beets, R. Glenn Weaver, Marc Davis, Christine Innes-Hughes, Anthony D. Okely

**Affiliations:** 1 School of Medicine, Science Medicine and Health, University of Wollongong, Wollongong, NSW Australia; 2 Illawarra Health and Medical Research Institute, University of Wollongong, Wollongong, NSW Australia; 3 Health Promotion Service, Illawarra Shoalhaven Local Health District, Warrawong, NSW Australia; 4 Early Start, School of Health and Society, University of Wollongong, Wollongong, NSW Australia; 5 Health Promotion Service, South Western Sydney Local Health District, Liverpool, NSW Australia; 6 Exercise Science, Arnold School of Public Health University of South Carolina, Columbia, SC USA; 7 Centre for Population Health, St Leonards, NSW Australia

**Keywords:** Healthy eating, Food environment, Nutrition, Dietary guideline, Out of school hours care, Afterschool care, Child care, Primary-school children

## Abstract

**Introduction:**

Out of school hours care (OSHC) is a fast-growing childcare setting in Australia, however the types of foods and beverages offered are relatively unknown. This study describes the food and beverages offered and investigates sector-level and setting-level factors which may impact OSHC in meeting the Australian Dietary Guidelines (ADG).

**Methods:**

This cross-sectional, observational study was conducted in 89 OSHC services (between 2018 and 2019). Food and beverages offered, kitchen facilities and menus were captured via direct observation. Foods were categorised into five food groups or discretionary foods, based on the ADG, and frequencies determined. Short interviews with OSHC directors ascertained healthy eating policies, staff training, food quality assessment methods and food budgets. Fisher’s exact test explored the influence of sector-level and setting-level factors on food provision behaviours.

**Results:**

Discretionary foods (1.5 ± 0.68) were offered more frequently than vegetables (0.82 ± 0.80) (*p* < .001), dairy (0.97 ± 0.81) (*p* = .013) and lean meats (0.22 ± 0.54) (*p* < .001). OSHC associated with long day care and reported using valid food quality assessment methods offered more lean meats (*p*= .002, and *p*= .004). Larger organisations offered more vegetables (*p* = .015) and discretionary foods (*p*= .007). Menus with clearly worded instructions to provide fruits and vegetables daily offered more fruit (*p*= .009), vegetables (*p* < .001) and whole grains (*p*= .003). No other sector or setting-level factors were associated with services aligning with the ADG.

**Conclusion:**

Future interventions could benefit from trialling menu planning training and tools to assist OSHC services in NSW meet the ADG requirements.

## Introduction

The vast majority of Australian children do not meet the national recommendations for consumption of vegetables (99.6%) or lean meats and meat alternatives (99.3%); and girls under-consume dairy products (96.1%) [1]. Furthermore, Australian children receive almost 40% of their energy from discretionary foods and beverages, high in saturated fat, salt or sugars [1, 2]. Poor dietary intake during childhood is likely to progress into adulthood and is linked to an increased risk of obesity and disease [3, 4]. Research has identified the important role that school and childcare services can play in fostering healthy food environments and promoting healthy eating practices in children [5, 6]. A number of interventions have focused on Australian schools and early childhood education and care settings (0-5 years) [6–9]. Less attention, however, has been given to food environments within the out of school hours care (OSHC) setting for primary school aged children (5-12 years). Although, studies conducted within Afterschool programs across the United States of America have frequently reported less than optimal food environments, with many services not achieving healthy eating standards [10–12].

OSHC is the second largest childcare setting in Australia, with children spending an average of 11 hours per week in OSHC [13]. The number of child enrolments have increased substantially from 162,000 in 2002, to 453,850 children in 2018 [13], with New South Wales (NSW) recording the highest proportion of child enrolments (144,140, 32%) in Australia [13].

While attending OSHC, children are provided with food and beverages (breakfast, morning or afternoon tea) by the OSHC service. Childcare in Australia is governed by the Australian Children’s Education and Care Quality Authority to ensure that child education and care settings meet the National Quality Framework and its seven National Quality Standards [14]. However, the only directive regarding the quality of food and beverages is that all food provided should be consistent with the Australian Dietary Guidelines (ADG) and water should always be available. The ADG are whole day guidelines which recommend consumption of a variety of foods from the five food groups (fruit, vegetables, grains, lean meats or alternatives and dairy) and to limit discretionary foods. Providing foods that align with the ADG can assist children to meet their daily nutritional requirements, especially of under-consumed food groups (e.g. vegetables) [15]. However, a part from this, no sector-specific guidelines exist.

Data relating to the types of food and beverages offered in OSHC services is lacking, with the most recent Australian studies conducted prior to 2003 [16, 17] and none have explored potential environmental factors that may be associated with providing healthy food options. As attendance in OSHC is growing [13], it is important to understand what foods and beverages are available for children and what sector and setting-level factors may influence the availability of healthy foods.

A socio-ecological model is a useful framework when exploring food environments as it accounts for the complex connection between sectors (e.g. government policy and legislation), settings (e.g. childcare), and individual factors that impact upon a person’s food choices and consumption behaviours [18]. For the purpose of this study, the focus is on connections between the food environment with sectors and settings.

This study aimed to 1) describe the types of foods and beverages offered within OSHC afterschool settings in two local health districts in New South Wales (NSW), Australia, and 2) examine how foods provided by services differed by sector-level and setting-level factors.

## Methods

### Study design and setting

A cross-sectional observational study was undertaken in OSHC services operating in the afterschool period (15:00 - 18:00) across two local health districts in NSW, Australia. The two districts contain metropolitan, suburban and rural communities and a diverse range of socioeconomic areas [19, 20]. This study was conducted according to the guidelines of the declaration of Helsinki, and approval was granted by the University of Wollongong Human Research Ethics Committee (HE17/490). The reporting of this research was guided by the Strengthening the Reporting of Observational Studies in Epidemiology (STROBE) checklist [21].

### Sample selection and recruitment

OSHC services from within the two districts were eligible to participate if they: operated from 15:00 - 18:00 during school terms; had a minimum of five primary school-aged children (5-12 years) enrolled each day; provided at least one afternoon snack; and were not exclusively advertised as a homework or physical activity-related club (e.g. dance academy or football club). Written informed consent was obtained from OSHC directors. OSHC service staff and parents were notified of the study via OSHC internal communication avenues and all information relating to this study was displayed at each OSHC service for a minimum of two weeks prior to data collection and during the data collection period. A detailed methodology has been previously published [22].

### Data collection

All data were collected between March 2018 to April 2019 by trained data collectors. This training included classroom simulation and practical on-site training at a local, non-participating OSHC service. Data were collected from each participating OSHC service on two, non-consecutive weekdays.

#### Food and beverage Observation and Categorisation

The types of food and beverages served to children were recorded via direct observation by trained data collectors, following previously published protocols [12, 23, 24]. Within this study, foods observed were reported as *offered*, rather than *consumed*, as the purpose of this study was to describe the types of foods served to children within the OSHC afterschool setting. Prior to foods being offered to children, they were recorded and photographed by a data collector. If foods were made prior to our arrival (e.g. cooked meals or prepared sandwiches) recipes were collected and all available nutritional labels were documented and photographed. Following this, food items were coded into the five food groups of the ADG [4, 25] with an additional sixth group for discretionary food items. Food groups were coded dichotomously, as offered or not offered and each food group was sub-categorised. For sub-categories, see Table 1.

**Table 1:** Food and beverages provided by OSHC services over a two day observation period

Food Description		Days (%) food & beverages were observed to be offered (n =176)
**Fruit**		**166 (94)**
Fresh fruit		165 (94)
Dried fruit		8 (5)
Canned fruit		6 (3)
**Vegetables**		**77 (44)**
Fresh/ raw		67 (38)
Cooked vegetables in meals		10 (6)
**Dairy or alternatives**		**90 (51)**
Cheese		66 (37)
Milk		16 (9)
Light milk		8 (5)
Full cream milk		8 (5)
Dairy alternatives		3 (2)
Yoghurt		15 (9)
Flavoured (full fat)		5 (3)
Flavoured (reduced fat)		10 (6)
**Lean meats or alternatives**		**19 (11)**
Beef or chicken		8 (5)
Chickpeas/hummus/baked beans		8 (5)
Eggs		1 (1)
Tuna		2 (1)
**Grains**		**124 (70)**
High fibre/whole grain		41 (23)
Refined grain		88 (50)
**Discretionary foods**		**142 (81)**
^ a^Processed meats		48 (27)
^ b^High salt/ low fibre snacks		50 (28)
^ c^Sweet snacks		32 (18)
^ d^Confectionary		52 (29)
^ e^Discretionary dairy		16 (9)
**Sauces and spreads**		
Sauces (Tomato / Barbecue / sweet chilli)		37 (21)
Cream cheese		23 (13)
Margarine		50 (28)
**Beverages**		
Water		174 (99)
100% fruit juice		2 (1)
Fruit drink		2 (1)
Milo™ (chocolate drink)		4 (2)

Foods may not have been provided in isolation, but with a combination of other reported items

*OSHC* out of school hours care

^a^ includes chicken nuggets, sausages, hotdogs/frankfurts, chorizo, luncheon meat, salami, cabanossi

^b^ includes two-minute noodles, chips, savoury biscuits >1800kJ/100g, packet soup mix, savoury pastries

^c^ includes chocolate, cakes, muffins, sweet biscuits/ cookies, jelly, muesli bars, sweet pastries

^d^ includes jam, honey, cinnamon sugar, sprinkles/ hundreds-and-thousands

^e^ includes cream, ice-cream, custard, butter, frozen yogurt

#### Sector-level factors

The Australian Bureau of Statistics, Socio-Economic Indexes for Areas, was used to classify each service into tertiles as being within a low, medium or high socio-economic area [26].

#### Setting-level factors

A brief, semi-structured interview was conducted with each OSHC service director during one of the site visits. The interview explored the service’s healthy eating policies and practices and was guided by the Healthy Afterschool Activity and Nutrition Documentation tool [27]. If the service had a healthy eating policy a copy of the policy was requested. Policies were assessed and the level of detail was categorised as: non-specific (limited detail, only states foods will be offered that align with the ADG) [25] or specific (clear objectives e.g. serve a fruit and vegetable each day, beverages will only include water and milk). Practices that were assessed included a) annual staff nutrition training: no training (<1 hour per year) or training (≥1 hour per year); b) the use of food assessment methods to assess weekly menus against the ADGs: non-valid assessments (no or limited assessment) or valid assessments (dietitian or use of a nutrition calculator); and c) grocery expenditure which was divided by the number of children per day to calculate daily expenditure.

In addition to exploring service’s policies and practices, we also observed food preparation facilities and menus. The types of facilities were coded as either: limited (sink, refrigerator, limited bench space and food storage space); moderate (sink, refrigerator, microwave, moderate bench space and food storage space); or complete (sink, refrigerator, microwave, oven, stove, dishwasher, large bench space and food storage space).

Menus were photographed on each observation day. Although no menu collected contained specific instructions or a checklist to offer all five food groups, a portion of menu templates contained instructions or a checklist component to serve fruit, or fruit and vegetables daily. Therefore, for the purpose of this analysis, menus were reviewed regarding their inclusion of fruits and vegetables and were coded as: none (no food groups mentioned), non-specific (serve a fruit or a vegetable daily) and specific (serve a fruit and a vegetable daily).

### Data analysis

Data were summarised using descriptive statistics, including frequency, mean (standard deviation (SD)) and median for food and beverages observed to be offered across two observation days. Shapiro-Wilk tests indicated that food data were skewed. A McNemar test was applied to explore if discretionary foods were offered more frequently than each of the five food groups (vegetables, fruit, grains, lean meats or meat alternatives, and dairy) with the McNemar-Bowker’s test of symmetry used to investigate differences between site visits. For categories that showed significance, a chi-square (McNemar test) was used to determine on which days the significant difference occurred. To control for multiple t-tests, a *p* value of < 0.017 was applied. Fisher’s Exact test was used to explore whether foods offered that aligned with the ADG differed across sector and setting-level factors. An alpha level of *p* < 0.05 was used for these analyses. All analyses were conducted using SPSS software (version 24, IBM Corporation, Armonk, NY, USA). Data were analysed in 2019-2020.

## Results

**Fig. 1:**
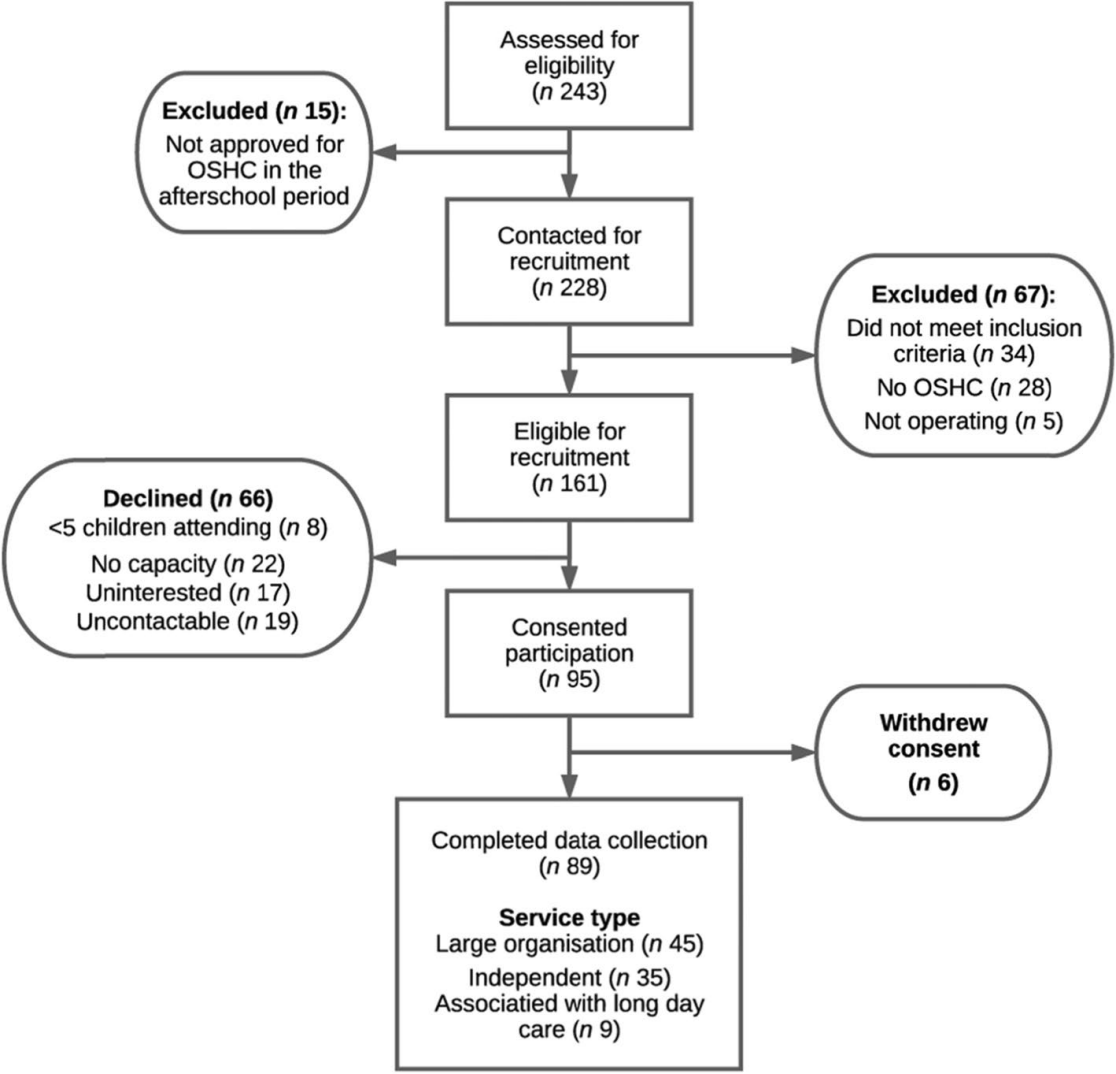
A flow diagram on recruitment of Out of School Hours Care (OSHC) services

There were a total of 243 OSHC services in the two Local Health Districts at the time of recruitment, a flowchart of the recruitment process and categorisation of service types has been presented (Fig. 1). In total, 89 OSHC services (37%) participated in the study with 4,408 children in attendance across the two observation days. All services were privately owned and located within school grounds (73%), community halls (17%) and early childhood settings (e.g. long day care) (10%). All services ran between 15:00 and 18:00, on at least four days a week and provided an afternoon snack to children.

### Food groups and meal types offered

Fruit was the most frequently observed food group offered as part of the afternoon snack (1.82 ± 0.47), followed by discretionary foods (1.5 ± 0.68), refined grains (0.98 ± 0.78), dairy (0.97 ± 0.81), vegetables (0.82 ± 0.80), whole grain (0.45 ± 0.75) and lean meats (0.22 ± 0.54). Findings from the McNemar-Bowker’s test indicate that discretionary foods were offered more frequently than vegetables (*p<* 0.001), dairy (*p* = .013) and lean meats or their alternatives (*p<* 0.001).The most commonly provided meal types consisted of fruit platters, sandwiches with confectionary fillings, healthy cooked meals and discretionary cooked meals (Table 2).

**Table 2:** Proportion (%) of meal type provided to children attending OSHC in the afterschool period.

Meal type	Description	Freq. (%) of meals observed
**Cooked meals**		
	Healthy	42 (24)
	Discretionary	25 (14)
**Sandwiches**		
	Confectionary	47 (26)
	Lean meat or alternative	13 (7)
	Salad	10 (6)
**Platter**		
	Fruit	51 (29)
	Fruit & Vegetable	20 (11)
	Savoury	21 (12)
**Other**		
	Dessert	8 (4)
	Fruit & Dairy	19 (11)

Healthy cooked meals include (pasta, rice, curry/ stir-fry)

Discretionary cooked meals include (chicken nuggets, sausage/ hotdog, pasties/ pies/ pizza scroll, two-minute noodles)

Confectionary sandwiches include (fillings of jam/ honey/ sprinkles/ hundreds and thousands)

Meat based sandwiches include (fillings of tuna, chicken breast, eggs)

Savoury platters include (could include a mixture of biscuits, dips, processed meats (cabanossi), cheese or vegetable sticks)

Dessert include (cakes, muffins, slices, sweet biscuits, jelly,)

Fruit & Dairy include (fresh or canned fruits with yogurt or custard)

*OSHC* out of school hours care

### Sector and setting level factors

Fifty-seven services (59%) provided their nutrition policy. Thirty-three services (37%) were part of a larger organisation and used the policy of the overarching organisation; therefore, 24 unique policies were collected. All policies were very similar, using non-specific language throughout their documentation and, therefore, were excluded from Fisher's Exact Test.

Table 3 presents findings from the Fisher’s Exact Test. OSHC services operating out of a long day care facility and those who reported assessing the quality of their menus with valid methods (nutrition calculator or dietitian) offered more lean meats or alternatives (*p *= .002, and *p*= .004 respectfully). OSHC associated with large organisations offered more vegetables (*p = *0.015) and discretionary foods (*p = *0.007). OSHC services that had menus which specified serving “fruit AND vegetables” daily, were observed to offer more fruits (p = 0.009), vegetables (*p *< 0.001) and whole grains (*p = *0.003)

**Table 3:** Differences in the provision of foods aligning with ADG by sector and setting level factors

.Service Characteristics	^**a**^Fruit (%)	^**b**^Vegetable (%)	^**c**^Dairy/ alternatives (%)	^**d**^Lean meats/ alternatives (%)	^**e**^Refined Grains (%)	^**f**^Whole grain (%)	^**g**^Discretionary (%)
**SECTOR -LEVEL**							
**SEIFA ranking**							
Low (n = 40 )	95	55	62	15	30	20	85
Medium (n = 31 )	96	70	80	20	43	43	97
High (n = 18 )	100	44	50	20	39	28	90
**SETTING-LEVEL**							
**Service Type**							
Large organisation (n = 43)	100	72*	70	11	49	35	98*
Independent (n = 37)	92	40	62	14	27	27	87
Long day care (n = 9)	100	62	62	63*	12	12	62
**Kitchen Facilities**							
Limited (n = 14)	100	57	71	0	100	43	93
Moderate (n = 21)	100	75	65	15	80	15	85
Complete (n = 54)	94	52	65	22	87	32	91
**Staff Training**							
No training (n = 59)	98	60	66	17	36	31	88
Training (n = 30)	93	53	67	17	37	27	93
**Daily cost of food AUD$**							
≤ $0.39 (n = 34)	94	59	62	12	47	41	94
$0.40 – $0.69 (n = 34)	100	49	63	15	24	27	85
≥ $0.70 (n = 21)	95	71	76	29	38	14	91
**Food Quality Assessment**							
None (n = 44)	100	63	60	9	42	33	88
Non-valid (n = 36)	94	50	69	17	25	19	89
Valid (n = 9)	89	67	78	56*	56	56	100
**Menu**							
None (n = 14)	84*	47	53	26	84	21	79
Non-specific (Fruit OR vegetable) (n = 44)	100	39	63	21	68	16	92
Specific (Fruit AND vegetable) (n = 31)	100	87*	77	7	58	52*	93

Socio-Economic Index for Areas (SEIFA)

^a^ Includes all fresh, frozen, canned in natural juice (not syrup). Excludes dried fruit and fruit juices

^b^ Includes vegetables that are fresh, frozen, cooked or canned

^c^ Includes fish, eggs, lean meat and poultry, nuts, seeds, legumes and beans

^d^ Includes milk, cheese, yoghurt, milk alternatives (calcium fortified alternatives). Excludes cream, sour cream, dairy desserts or iced confectionary (ice cream or frozen yoghurts)

^e^ Includes all grains, bread, cereals, rice, pasta, noodles, couscous and polenta

^f^ Includes all grains products specified as whole grain, whole meal, rye, barley, oats and quinoa.

^g^Includes cream, sweet biscuits, cakes, pastries, pies, processed meat, chips or savoury crackers >1800kJ/100g, high sugar/ salt/ fat spreads, sugar-sweetened beverages and lollies/ candy.

*ADG* Australian Dietary Guidelines

* Indicates values are significant *p* < 0.05

## Discussion

This cross-sectional study observed the food and beverages provided to children (5-12 years), across a large sample of OSHC afterschool services. To the authors’ knowledge, this is the first observational study to explore both the food and beverages offered and sector-level and setting-level factors that may influence compliance with ADG within Australian OSHC services. We found fruit was the most common food group offered across all observation days, however discretionary foods were observed significantly more than vegetables, dairy and lean meats or their alternatives. Water was the most frequent beverage type offered. Results from Fisher’s Exact Test indicate a number of environmental factors were found to be associated with offering food groups aligning with the ADG.

Discretionary foods are recommended to be consumed sometimes and in small amounts [25], yet our results indicate that discretionary foods may frequently be offered by OSHC services. Although the Guidelines are an important national resource, their appropriateness as the sole resource for the OSHC setting may be unsuitable as before-school and after-school services only provide breakfast or an afternoon snack across a child’s day. To consider this within the context of a child’s day, findings from the National Health Survey reports that discretionary foods, specifically cakes, sweet biscuits and processed meats, are some of the primary sources of energy, saturated fats, added salt and sugars within children’s diet [28]. Additionally, a NSW study reported children have on average, 1.5 serves of discretionary foods already within their school lunch boxes [29, 30]. As Australian children may be exceeding recommended serves of discretionary foods outside of the OSHC setting, it highlights the need for clear healthy eating guidelines specific to the OSHC sector, especially regarding discretionary foods. An example of how clear guidelines and policy within the school setting may have had a positive influence on the OSCH sector, can be seen by comparing beverage data before and after the introduction of the mandatory cessation of the sale of sugar sweetened beverages in NSW Government schools in 2007 by the NSW Government. Data collected in OSHC services prior to this date reports 24% of services offering sugary beverages (cordial) to children [17], in comparison to just 1% of services in the present study.

Other childcare settings, such as Early Childhood Education and Care, have clear sector-specific guidelines (“Caring for Children - birth to 5yrs*”*) [31], to assist their services to offer foods consistent with the ADG [25], Infant Feeding Guidelines [32] and the National Quality Standards [14]. *“*Caring for Children - birth to 5 years*”* contains a detailed menu planning section outlining the type and quantity that each food group should provide each day. Serving lean meats or  meat alternatives daily is one such recommendation. This recommendation  may have indirectly impacted the behaviour of OSHC services associated with long day care (primarily a care setting for 0-5years) and may explain why these services were observed to offer significantly more lean meats within our study; as a portion of the lunch time meal was provided during the afternoon OSHC. Further to this, the use of valid food quality assessment methods (dietitian or nutrition calculator), at least once a year, was also positively associated with services offering more lean meats or their alternative. As lean meats were seldom observed within our sample, the provision of annual menu support, such as dietitian or nutrition calculator, may assist support OSHC services to make achievable improvements to their menu practices and provide foods that align with dietary guideline recommendations.

Although our findings demonstrate OSHC services are regularly providing fruit; vegetables were observed on less than half the observation days. Interestingly, we found significantly more vegetables and, surprisingly, whole grains to be offered at services that used menu planning template with a checklist instructing the provision of “fruit and vegetables” daily. These types of menu planning templates were found mostly in OSHC services associated with large organisations, who disseminated a uniform menu planning template across all of their services and may explain why these services were more likely to offer vegetables. It is however, unclear why whole grains were associated with menu planning templates specifying to offer “fruit and vegetables” and may be a chance finding. Although uniformity in menu templates can equate to positive behaviours such as serving more vegetables, we found that the opposite was also possible. Large organisations in this sample were also linked to offering discretionary foods more frequently, which may be due to organisations within our study using an identical daily menu across each of their services, with their daily menu consisting of sandwiches; processed meat, cream cheese, jam and honey with a fruit and vegetable platter. Producing menus compliant with dietary guidelines has been identified throughout the literature as a complex task [33] with a number of key barriers including, a lack of training, resources and ongoing support [34–36].

Evidence from systematic literature reviews indicates that in order to make significant behaviour changes, and to support menu development to align with dietary guidelines, multi-component interventions are needed within childcare settings [5, 35]. A randomised controlled trial, conducted in NSW, within the early childhood education and care sector applied a multicomponent intervention focusing on: staff training (menu planning workshop), menu audits and feedback, face-to-face support and additional resources (menu planning template/checklist) [37]. The intervention found a significant improvement in recommended food groups on planned menus compared to the control group, and a significant increase in child vegetable and fruit consumption was evident in the intervention group [37]. Although nutrition training was not found to be associated with food groups served within our study, this may be due to the type, quality and frequency of the training provided. Currently in NSW, there is no tailored nutrition training, menu development support or feedback available to OSHC services and therefore any training provided to OSHC staff would have been organised internally and may not have been sufficient to produce behaviour change. Future interventions should trial the effect of sector-specific guidelines, nutrition training and menu planning tools to support effective behaviour changes within the OSHC setting.

The findings in our study need to be considered in context of its limitations. Firstly, although this study sample included a number of services from a diverse geographical landscape, all services were recruited from within two local health districts in NSW and may not be representative across NSW or Australia. Secondly, this study observed food groups provided by OSHC and did not report on the number of servings per child nor actual consumption of food and beverages. Finally, there is potential that some self-reported data may have been misreported, as desirable practices rather than actual practices.

## Conclusion

Findings from this study indicate that OSHC services in NSW may not be providing foods in accordance with ADG, specifically for vegetables, lean meats and their alternatives, dairy and discretionary foods. Introducing a menu planning tool specific to the OSHC setting may be a useful and cost-effective resource to provide a variety of food groups aligning with the dietary guidelines. Future research should focus on the impact of multiple-component interventions (such as the development of sector-specific guidelines, training opportunities, and menu planning tool) on the quality of foods offered within NSW OSHC services.

## Data Availability

The datasets generated and analysed during the current study are not publicly available but are available from the corresponding author on reasonable request.

## Abbreviations

ADG: Australian Dietary Guidelines
OSHC: Out of school hours care
NSW: New South Wales
